# BLACK PLACEMAKING: THE BODY, HOME, AND PUBLIC SPACE THROUGH THE LENS OF OLDER WOMEN

**DOI:** 10.1093/geroni/igac059.809

**Published:** 2022-12-20

**Authors:** Laurent Reyes, Jarmin Yeh, H Shellae Versey

**Affiliations:** UC Berkeley, Berkeley, California, United States; University of California, San Francisco, San Francisco, California, United States; Fordham University, Bronx, New York, United States

## Abstract

African American communities are frequently depicted as victims of urban conditions. However, a rich culture of grassroots community development and organizing, often led and stewarded by Black women, exists. Many of these efforts involve enhancing economic, political, and educational opportunities and centering ethics of care and caregiving. This is the notion of Black placemaking, which is explicitly community-focused, shaping the social fabric of everyday life and allowing for the development of Black vernacular spaces that became vital to African-American culture. This paper examines how Black older women engage in placemaking by presenting three select case studies. Using a narrative inquiry approach, we conducted secondary data analysis of interviews drawn from larger qualitative studies about aging in communities that took place in San Francisco and New York City. Black feminist spatial imagination, embodiment, and intersectionality theory were our guiding frameworks. Our analysis revealed how the aging Black body is a site that is subjected to socio-political regulation and violence and illuminates how Black women are agents of community resilience, creativity, and transformation. Creating and holding space (i.e., placemaking) with bodies and physical structures that center the Black community is an act of care, self-determination, and resistance to white supremacy. These embodied processes of placemaking have wide-ranging implications for the ways Black neighborhoods are framed and discussed in popular media, empirical research, and policy. Furthermore, they invite a shift in our current approach to placemaking in later life, one that centers the strengths, history, and traditions of the Black community.

